# Adenylyl-Sulfate Kinase (Met14)-Dependent Cysteine and Methionine Biosynthesis Pathways Contribute Distinctively to Pathobiological Processes in Cryptococcus neoformans

**DOI:** 10.1128/spectrum.00685-23

**Published:** 2023-04-10

**Authors:** Seung-Heon Lee, Yu-Byeong Jang, Yeseul Choi, Yujin Lee, Bich Na Shin, Han-Seung Lee, Jong-Seung Lee, Yong-Sun Bahn

**Affiliations:** a Department of Biotechnology, College of Life Science and Biotechnology, Yonsei University, Seoul, Republic of Korea; b AmtixBio Co., Ltd., Hanam-si, Gyeonggi-do, Republic of Korea; University of Michigan

**Keywords:** sulfur amino acids, sulfate assimilation pathway, Met14, Met3, virulence, fungal pathogens

## Abstract

Blocking of nutrient uptake and amino acid biosynthesis are considered potential targets for next-generation antifungal drugs against pathogenic fungi, including Cryptococcus neoformans. In this regard, the sulfate assimilation pathway is particularly attractive, as it is only present in eukaryotes such as plants and fungi, yet not in mammals. Here, we demonstrated that the adenylyl sulfate kinase (Met14) in the sulfate assimilation pathway is not essential yet is required for the viability of C. neoformans due to its involvement in biosynthesis of two sulfur-containing amino acids, cysteine and methionine. Met14-dependent cysteine and methionine biosynthesis was found to significantly contribute to a diverse range of pathobiological processes in C. neoformans. Met14-dependent cysteine rather than methionine biosynthesis was also found to play pivotal roles in cell growth and tolerance to environmental stresses and antifungal drugs. In contrast, the Met14-dependent methionine biosynthesis was found to be more important than cysteine biosynthesis for the production of major cryptococcal virulence factors of melanin pigments and polysaccharide capsules. Finally, we also found that despite its attenuated virulence in an insect model, Galleria mellonella, the *met14*Δ mutant yielded no difference in virulence in a murine model of systemic cryptococcosis. Hence, clinical inhibition of Met14-dependent amino acid biosynthetic pathways may not be advantageous for the treatment of systemic cryptococcosis.

**IMPORTANCE** Current antifungal drugs have several limitations, such as drug resistance, severe side effects, and a narrow spectrum. Therefore, novel antifungal targets are urgently needed. To this end, fungal sulfur amino acid biosynthetic pathways are considered potential targets for development of new antifungal agents. Here, we demonstrated that Met14 in the sulfate assimilation pathway promotes growth, stress response, and virulence factor production in C. neoformans via synthesis of sulfur-containing amino acids methionine and cysteine. Met14-dependent cysteine rather than methionine synthesis was found to be critical for growth and stress responses, whereas Met14-dependent methionine synthesis was more important for the production of antiphagocytic capsules and antioxidant melanin in C. neoformans. Surprisingly, deletion of the *MET14* gene was found to attenuate cryptococcal virulence in an insect model, yet not in a murine model. Collectively, our results showed that Met14-dependent cysteine and methionine biosynthesis play roles that are distinct from each other in C. neoformans. Moreover, Met14 is unlikely to be a suitable anticryptococcal drug target.

## INTRODUCTION

Sensing, uptake, and utilization of nutrients such as carbon, nitrogen, and sulfur are essential for the survival of microorganisms living in biotic or abiotic environments, and in particular for microbial pathogens that compete for the limited available nutrients within susceptible hosts (1). Sulfur is an essential primary nutrient for synthesizing amino acids such as methionine, cysteine, homocysteine, and taurine, and antioxidants, such as glutathione. Of the four sulfur-containing amino acids (SAAs), methionine and cysteine are essential for protein translation. Methionine is the first amino acid used in eukaryotic protein synthesis, whereas cysteine contributes to tertiary or quaternary protein structure formations by forming disulfide bonds with other cysteine residues. Glutathione is a reductive molecule synthesized from glycine, cysteine, and glutamate and plays a critical role in defense against reactive oxygen species (2), nutrient deprivation (3), and heavy metal stress (4). Hence, biosynthetic pathways of SAA are crucial for the survival of various bacterial and fungal pathogens within a host.

**FIG 1:**
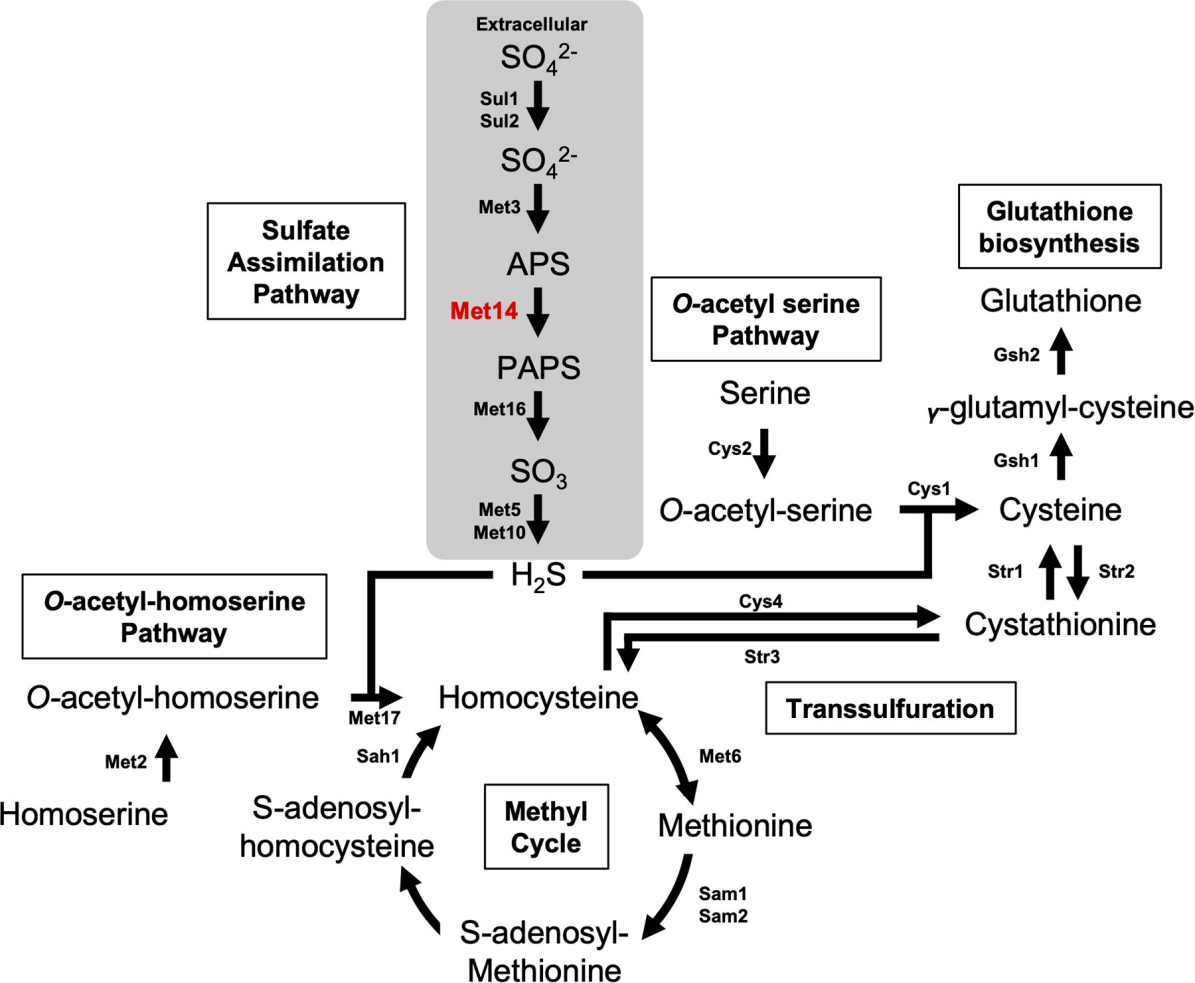
Proposed pathways for sulfur uptake and sulfur amino acid biosynthesis in fungi. Extracellular sulfate is converted to intracellular hydrogen sulfide in the sulfur assimilation pathway to synthesize cysteine and methionine through the OAS pathway, OAH pathway, methyl cycle, and transsulfuration pathway. Glutathione can be synthesized from cysteine through the glutathione biosynthesis pathway (6, 18, 54).

The sulfate assimilation pathway, in which ATP sulfurylase, adenylyl-phosphosulfate (APS) kinase, phospho-APS (PAPS) reductase, and sulfite reductases are involved, plays a pivotal role in converting sulfate to sulfide and thereby releases sulfur atoms for use in other sulfur-requiring processes (5) (Fig. 1). In most eukaryotes, except mammals, this sulfate assimilation pathway provides sulfide to two different SAA biosynthetic pathways, namely, the *O*-acetyl-homoserine (OAH) and *O*-acetyl-serine (OAS) pathways (6). In the OAH pathway, homoserine is converted to *O*-acetyl-homoserine, which is then converted to homocysteine using H_2_S (6). The synthesized homocysteine can be reversibly interchanged with methionine through the methyl cycle (7). In the OAS pathway, serine is converted to *O*-acetyl-serine, which is then converted to cysteine using H_2_S. The OAH and OAS pathways are connected to the transsulfuration pathway, which converts homocysteine first to cystathionine and then to cysteine (6).

Glutathione has a high reducing potential due to the thiol (SH) group of its constituent amino acid, cysteine. Following transsulfuration, γ-glutamyl-cysteine synthetase catalyzes conversion of cysteine to γ-glutamyl-cysteine, which is then converted to glutathione by glutathione synthetase (8). This tripeptide molecule serves not only as a strong antioxidant defense system but also as a nonprotein sulfur reservoir (9, 10). The sulfate assimilation pathway controls the intracellular glutathione concentration in Saccharomyces cerevisiae (11).

The role of SAA biosynthesis pathways in the virulence of invasive human fungal pathogens, such as Aspergillus fumigatus, Candida albicans, and Cryptococcus neoformans, has been studied previously. The sulfur assimilation pathway has also gained attention for antifungal drug development, since methionine is an essential amino acid that humans cannot synthesize *de novo*. Methionine must be acquired from the diet or microbiome in mammals (12). Aspergillus fumigatus requires a bZIP transcription factor involved in the sulfate assimilation pathway, MetR, for virulence and iron homeostasis, suggesting the presence of a link between sulfate assimilation pathway and iron homeostasis (13). The sulfate assimilation pathway of C. albicans is highly similar to that of Saccharomyces cerevisiae. Candida albicans Met15, an *O*-acetyl homoserine-*O*-acetyl serine sulfhydrylase, is required for glutathione production and virulence (14). Met3 is the first reported factor involved in the sulfur assimilation pathway of C. neoformans, which causes fatal meningoencephalitis in immunocompromised patients. In addition to its critical role in growth, Met3 is involved in melanin biosynthesis, thermotolerance, and virulence as well (15). Met3 was also recently reported as a core virulence-related kinase required for lung and brain infections by C. neoformans (16). The adenylyl-sulfate kinase (APS kinase) Met14 in the sulfur assimilation pathway was first reported to be a putative essential kinase in C. neoformans (17). However, Met14 has also recently been reported to be nonessential in Cryptococcus deneoformans, and a deletion mutant was found to show auxotrophism for SAAs (18).

Here, we performed a functional characterization of APS kinase (Met14) in C. neoformans, which is the most dominant pathogenic cryptococcal species isolated from natural and clinical environments. We found that deletion of the *MET14* gene was feasible in C. neoformans, indicating that it is not an essential kinase but leads to auxotrophic growth defects in SAAs. Interestingly, we also found that Met14 was not required for C. neoformans growth in nutrient-rich media, such as yeast extract-peptone-dextrose (YPD) medium. However, Met14 had significant effects on stress responses, adaptation, and virulence factor production. Most of these mutant phenotypes likely resulted from significantly reduced SAA and glutathione production in the *met14*Δ mutant. Unlike its upstream kinase Met3, Met14 was also not required for the virulence of C. neoformans in a murine model of systemic cryptococcosis, even though perturbation of Met14 was found to reduce virulence in an insect model. Our findings provide novel insights into the fungal sulfur assimilation pathway and highlight that not every enzyme involved in amino acid biosynthesis or sulfur metabolism is required for virulence.

## RESULTS

### Met14 is a nonessential kinase involved in SAA biosynthesis in C. neoformans.

**FIG 2:**
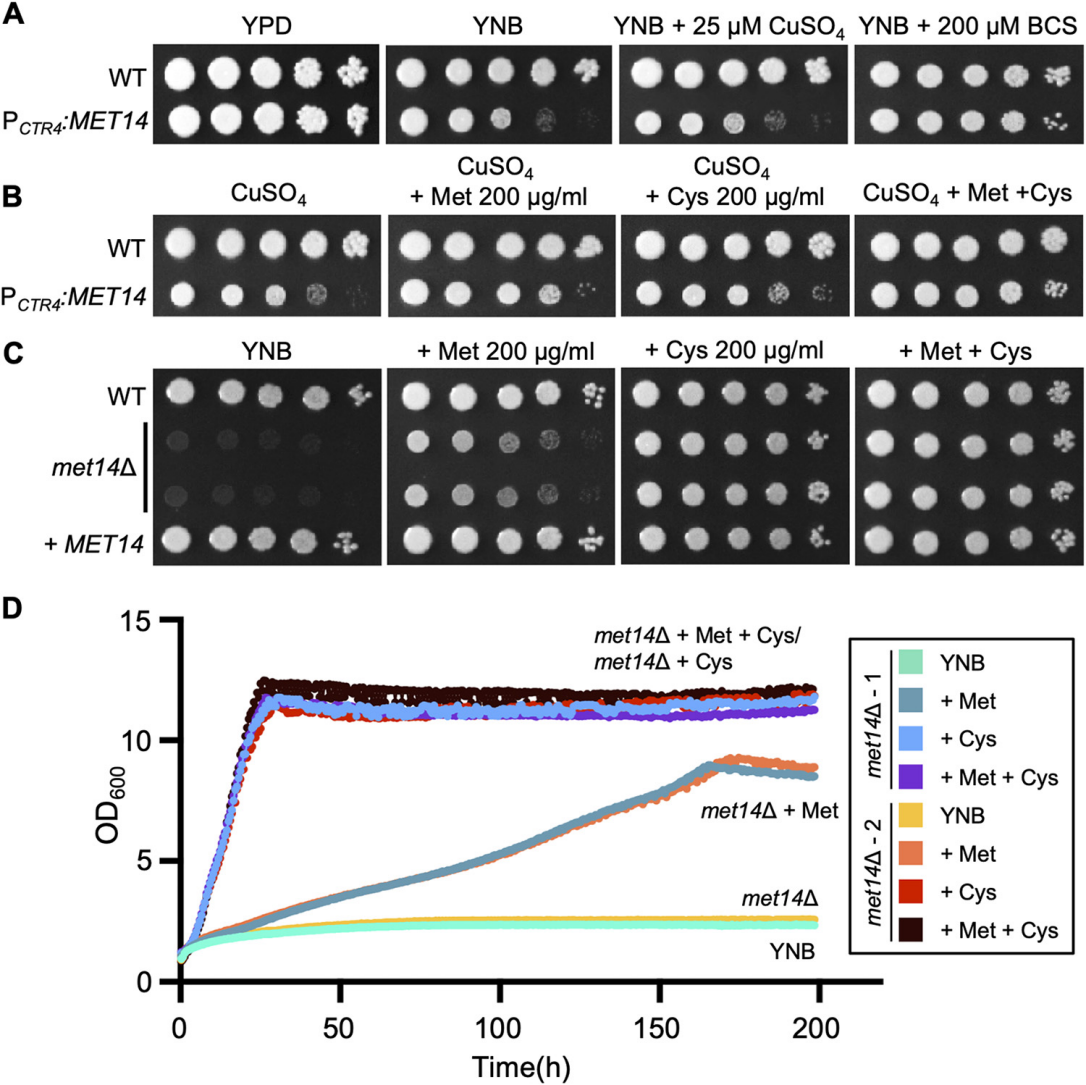
Met14-dependent sulfur amino acid biosynthesis is required for the growth of C. neoformans. (A) Cryptococcal growth inhibition by repression of *MET14*. The wild-type (WT; H99) and P*_CTR4_*:*MET14* (YSB5818) strains were cultured overnight at 30°C in liquid YPD medium, serially diluted (1 to 10^4^), and spotted onto nutrient-rich YPD or nutrient-defined YNB plates containing 25 μM CuSO_4_ for repression of *MET14* or 200 μM BCS for induction of *MET14*. The plates were incubated at 30°C for 3 days and photographed. (B) Growth complementation of P*_CTR4_*:*MET14* under repressive conditions with the addition of methionine and/or cysteine. Cells prepared as for panel A were spotted onto the YNB plates containing 25 μM CuSO_4_ with or without the indicated amount of Met and/or Cys. The plates were incubated in a 30°C incubator for 3 days and photographed. (C) Methionine and cysteine auxotrophic growth of *met14*Δ. The wild-type (WT; H99), *met14*Δ (YSB6851 and YSB6852), and *met14*Δ::*MET14* complemented (+*MET14*; YSB10248) strains were cultured overnight at 30°C in liquid YPD medium, serially diluted (1 to 10^4^), and spotted on YNB (with ammonium sulfate) plates supplemented with methionine and/or cysteine. The plates were incubated at 30°C for 3 days and photographed. (D) Quantitative measurement of *met14*Δ growth with or without supplementation of methionine and/or cysteine. Growth was monitored at 30°C by measuring the OD_600_ with a multichannel bioreactor (SIA Biosan, Riga, Latvia) until the growth of *met14*Δ supplemented with methionine plateaued. Two independent *met14*Δ strains (YSB6851 #1 and YSB6852 #2) were monitored.

*MET14* was considered a putative essential kinase in our previous systematic functional analysis of C. neoformans kinases, since deletion mutants could not be generated (17). To investigate the role of *MET14* in growth and viability of C. neoformans, we generated a P*_CTR4_*:*MET14* strain in which the native *MET14* promoter was replaced with the *CTR4* (copper transporter 4) promoter, which is regulated by the concentration of copper ions (19) (see Fig. S1A in the supplemental material). In these strains, the addition of copper ions (CuSO_4_) to the growth medium was found to inhibit *MET14* expression, whereas the copper chelator bathocuproine disulfonic acid (BCS) was found to significantly induce *MET14* expression above its native levels (by 10-fold) (Fig. S1B). P*_CTR4_*:*MET14* showed no growth defects in the nutrient-rich YPD medium, whereas growth defects were observed in nutrient-minimal yeast nitrogen base (YNB) medium or YNB supplemented with CuSO_4_ (Fig. 2A). However, BCS addition was found to completely restore the normal growth behavior of P*_CTR4_*:*MET14* in the YNB medium (Fig. 2A), indicating that *MET14* is required for C. neoformans growth. Met14 in S. cerevisiae is part of the sulfur assimilation pathway required for the synthesis of cysteine and methionine. Hence, we next investigated the effect of addition of SAAs on growth of P*_CTR4_*:*MET14* in YNB + CuSO_4_ medium. As expected, the addition of methionine or cysteine was found to significantly restore the growth of P*_CTR4_*:*MET14* in YNB + CuSO_4_ medium (Fig. 2B).

Because P*_CTR4_*:*MET14* was found to grow in SAA-supplemented medium, we attempted to generate *MET14* deletion mutants using YPD medium supplemented with additional methionine and cysteine. We successfully generated two independent *met14*Δ strains, which were confirmed with Southern blot analysis (Fig. S2). Similar to P*_CTR4_*:*MET14*, *met14*Δ strains were also found to have a growth defect on YNB medium. Growth was rescued upon the addition of SAAs and reinsertion of the wild-type *MET14* gene (Fig. 2C). Addition of cysteine rescued growth of *met14*Δ more efficiently than did addition of methionine (Fig. 2C and D). Considering the critical roles of both methionine and cysteine for protein synthesis in C. neoformans, a cysteine-to-methionine conversion is likely more efficient than a methionine-to-cysteine conversion in C. neoformans. Collectively, these data indicated an involvement of Met14 in SAA biosynthesis, which is required for C. neoformans growth under minimal nutrient conditions.

### Met14 is essential for stress and antifungal drug responses in C. neoformans.

**FIG 3:**
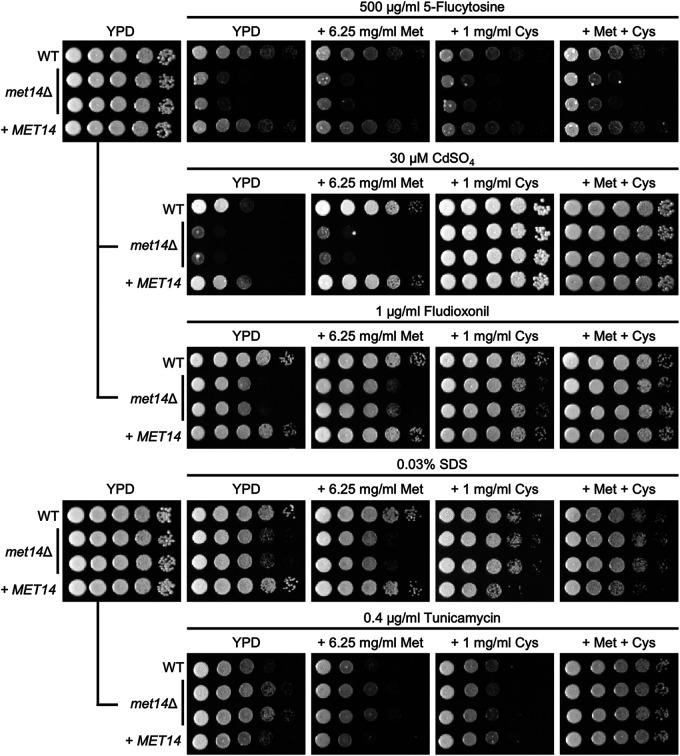
Met14-dependent cysteine biosynthesis is required for the stress response of C. neoformans. The wild-type (WT; H99), *met14*Δ (YSB6851 and YSB6852), and *met14*Δ::*MET14* complemented (+*MET14*; YSB10248) strains were cultured overnight at 30°C in liquid YPD, serially diluted (1 to 10^4^), and spotted onto the YPD plates containing 6.25 mg/mL methionine and/or 1 mg/mL cysteine and the indicated amount of stress or antifungal agents. Plates were incubated at 30°C and photographed from 1 to 4 days.

We next identified the susceptibility of *met14*Δ to several stress-inducing chemical reagents to investigate the role of Met14 in additional biological functions of C. neoformans. For this purpose, we used nutrient-rich YPD as a basal medium, since growth of *met14*Δ was found to be similar to that of wild-type in this medium. The *met14*Δ mutant was also found to be just as resistant to high temperature, antifungal drugs (amphotericin B and fluconazole), osmotic stresses (NaCl, KCl, and sorbitol), genotoxic stresses (hydroxy urea and methyl methanesulfonate), oxidative stresses (diamide, hydrogen peroxide, *tert*-butyl hydroperoxide, and menadione), endoplasmic reticulum (ER) stress (dithiothreitol), and cell wall stress (Congo red) (Fig. S3). However, the *met14*Δ mutant showed higher susceptibility to a toxic heavy metal (CdSO_4_), cell membrane stress agent (SDS), and some antifungal agents (5-flucytosine and fludioxonil), whereas it showed slight resistance against tunicamycin, which blocks *N*-linked glycosylation and induces the unfolded protein response in the ER (Fig. 3).

We hypothesized that several stress-related phenotypes observed in the *met14*Δ mutants may be due to insufficient levels of SAAs or their related metabolites, even under nutrient-rich conditions. A lower efficiency in protein translation owing to an insufficient supply of SAAs may also reduce the accumulation of toxic unfolded proteins caused by tunicamycin. We thus tested the susceptibility of *met14*Δ to the aforementioned stress agents by adding either methionine or cysteine (or both). We found that addition of cysteine rather than methionine to YPD restored wild-type resistance to CdSO_4_, fludioxonil, SDS, and tunicamycin (Fig. 3). Thus, an insufficient supply of cysteine, but not methionine, due to *MET14* deletion altered the resistance behavior of C. neoformans to these stress agents. Interestingly, the addition of neither methionine nor cysteine (or both) rescued wild-type resistance to 5-flucystoine (Fig. 3), suggesting that Met14 may have a unique role in defense against this DNA and RNA synthesis inhibitor in an SAA biosynthesis-independent manner.

### Met14-dependent glutathione synthesis contributes to stress response and adaptation in C. neoformans.

Our data described above indicated that Met14-dependent cysteine synthesis is more important than methionine synthesis in facilitating the growth and stress response of C. neoformans. Cysteine is not only a fundamental amino acid for protein synthesis but also is a major constituent of the antioxidant glutathione. Furthermore, heavy metal stress induced by cadmium sulfate and antifungal drug fludioxonil treatment may be linked to the intracellular glutathione level. Heavy metals may induce neurotoxicity, genotoxicity, and oxidative stress by disrupting prooxidant and antioxidant homeostasis (20). Fludioxonil, a phenylpyrrole fungicide, was recently reported to induce oxidative stress in various organisms (21–23). Hence, we next investigated the effect of *MET14* deletion on intracellular glutathione levels.

**FIG 4:**
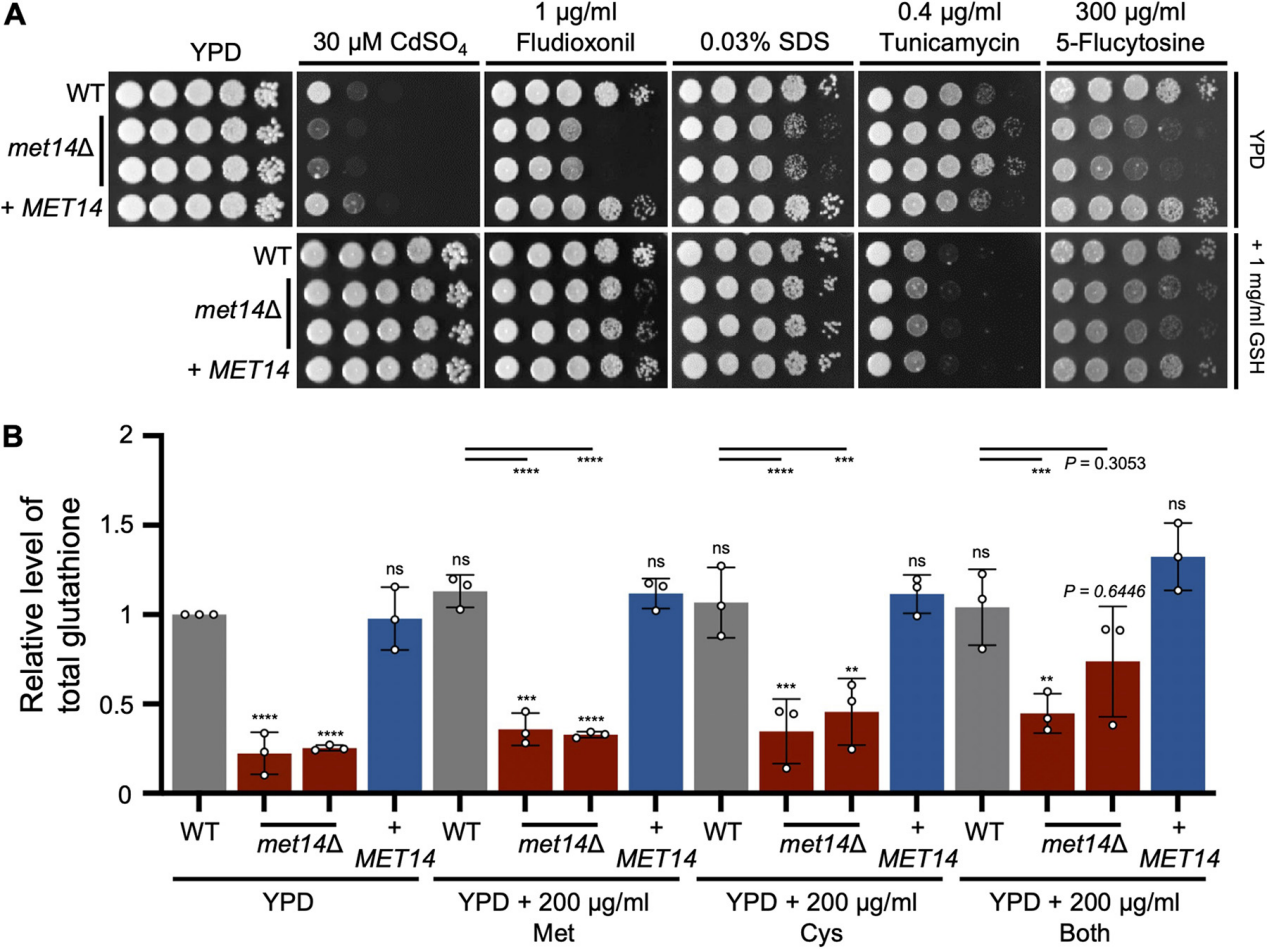
Met14 is required for glutathione synthesis of C. neoformans. (A) Normal growth and stress resistance restoration in *met14*Δ by glutathione supplementation. The wild-type (WT; H99), *met14*Δ (YSB6851 and YSB6852), and *met14*Δ::*MET14* complemented (+*MET14*; YSB10248) strains were cultured overnight at 30°C in liquid YPD medium, serially diluted (1 to 10^4^), and spotted onto the YPD plates containing the indicated amount of stress and antifungal reagents and with or without 1 mg/mL of exogenous glutathione. Plates were wrapped with aluminum foil to avoid light, incubated at 30°C, and photographed from 1 to 3 days. The images of YPD control, YPD + 0.03% SDS, and YPD + 0.4 μg/mL tunicamycin plates were reused, since this experiment was conducted in the same batch shown in the fourth and fifth rows of Fig. 3. (B) Quantitative measurement of relative total glutathione levels in *met14*Δ. Total glutathione levels (GSH and GSSG) of wild-type, *met14*Δ, and complemented strains grown in YPD with or without methionine and/or cysteine were assayed as described in Materials and Methods and normalized to dry cell weight. Relative level of total glutathione was calculated in comparison with the wild-type value. Three independent experiments were performed. Data represent means ± standard errors. Statistical significance of difference was determined by one-way analysis of variance (ANOVA) with Bonferroni’s multiple-comparison test: *, *P* < 0.1; **, *P* < 0.01; ***, *P* < 0.001; ****, *P* < 0.0001; ns, not significant.

First, we tested whether glutathione supplementation could restore the wild-type resistance of *met14*Δ to stress-inducing agents (Fig. 3). Similar to cysteine supplementation, glutathione supplementation almost completely recovered the wild-type resistance of *met14*Δ to CdSO_4_, SDS, fludioxonil, and tunicamycin, but only partially to resistance to 5-flucytosine (Fig. 4A). Interestingly, addition of SAAs or glutathione was found to significantly increase the resistance of the wild-type strain to CdSO_4_ (Fig. 3 and 4A). This indicated that SAA biosynthesis is vital for the response to heavy metal stress.

Next, we investigated whether glutathione addition could restore stress resistance via its antioxidant activity *per se* or as a cysteine source. Glutathione exists in two different molecular forms in the cell, reduced glutathione (GSH) and oxidized glutathione (GSSG). GSH reduces oxidative molecules inside the cellular environment to GSSG. GSSG can then be recycled back to GSH by glutathione reductase. Glutathione can be degraded by γ-glutamyl transpeptidase (24). Since the sulfate assimilation pathway controls glutathione synthesis upstream, we reasoned that *met14*Δ may display a lower glutathione concentration. Indeed, total glutathione levels in *met14*Δ were significantly lower than those in the wild-type and complemented strains under basal conditions (Fig. 4B). However, addition of SAAs was not found to significantly increase total glutathione levels in either the wild-type or *met14*Δ strains (Fig. 4B). Moreover, glutathione supplementation was even found to rescue growth of the *met14*Δ strain in minimal YNB medium (Fig. S4). Altogether, these results indicated that glutathione addition can rescue the normal growth and stress responses of *met14*Δ by providing cysteine, rather than by its antioxidant activity.

### Met14-dependent methionine synthesis rather than cysteine synthesis is required for capsule and melanin production.

Next, we investigated the role of Met14 in the production of capsules and melanin, two major virulence factors in C. neoformans. Melanin is an amorphous brownish antiphagocytic pigment that provides defense against temperature shifts and reactive oxygen species (25). Polysaccharide capsules on the outermost surface layer protect C. neoformans from phagocytosis by the host immune system. Capsule enlargement also enhances resistance against oxidative stress (26).

**FIG 5:**
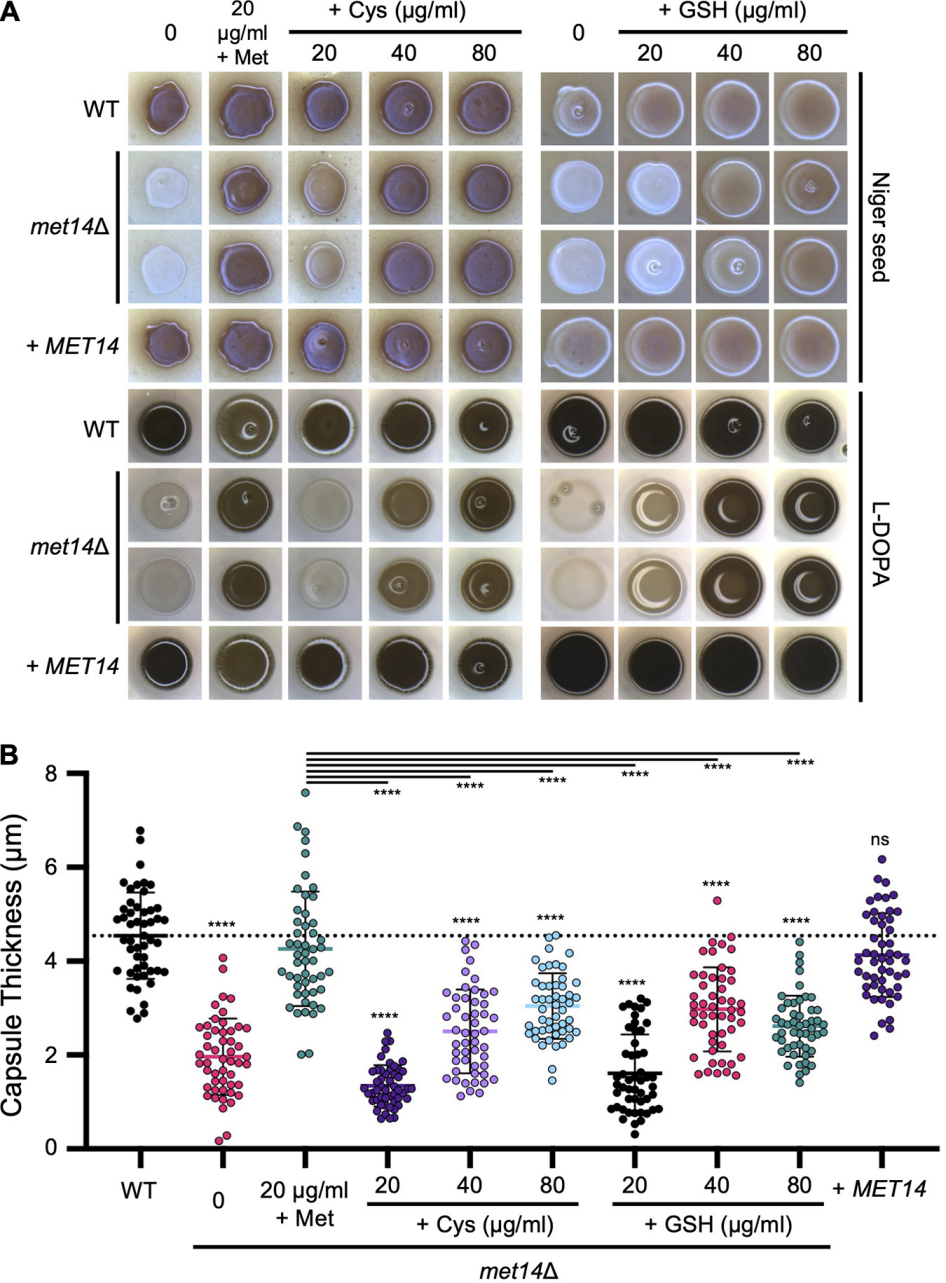
Met14-dependent methionine plays a major role in the production of capsule and melanin. (A) Melanin induction assay of wild-type and *met14*Δ mutant strains on Niger seed and l-DOPA media. The indicated amounts of methionine, cysteine, or glutathione were added to the melanin induction medium. Darker colony color indicates greater induction of melanin pigment than the lighter colonies. (B) Capsule induction assay of wild-type and *met14*Δ mutant strains on Littman’s medium containing the indicated amount of methionine, cysteine, or glutathione. Each measurement was repeated for 50 cells under each condition as indicated. Error bars indicate standard deviations. Statistical significance of differences was determined by one-way ANOVA with Bonferroni’s multiple-comparison test using Prism 8.0: *, *P* < 0.1; **, *P* < 0.01; ***, *P* < 0.001; ****, *P* < 0.0001; ns, not significant.

*met14*Δ showed significantly reduced levels of melanin and capsule thickness compared to wild-type and complemented strains in melanin- and capsule-inducing media, respectively (Fig. 5A and 5B; Fig. S5 and S6). Surprisingly, unlike their effects on growth and stress response, methionine rather than cysteine addition recovered the production of melanin and capsule efficiently, as 20 μg/mL of methionine was found to completely recover wild-type levels of melanin and capsule, whereas the same concentration of cysteine did not have such an effect (Fig. 5A and B). Higher cysteine concentrations (40 to 80 μg/mL) almost completely restored melanin production, yet capsule production recovered only partially in *met14*Δ (Fig. 5A and B). Supplementation of GSH also resulted in similar findings (Fig. 5A and B). These data demonstrated that Met14-dependent methionine synthesis is essential for the synthesis of the major cryptococcal virulence factors, melanin and capsule.

### *MET14* is essential for C. neoformans virulence in an insect model, but not in an animal model.

**FIG 6:**
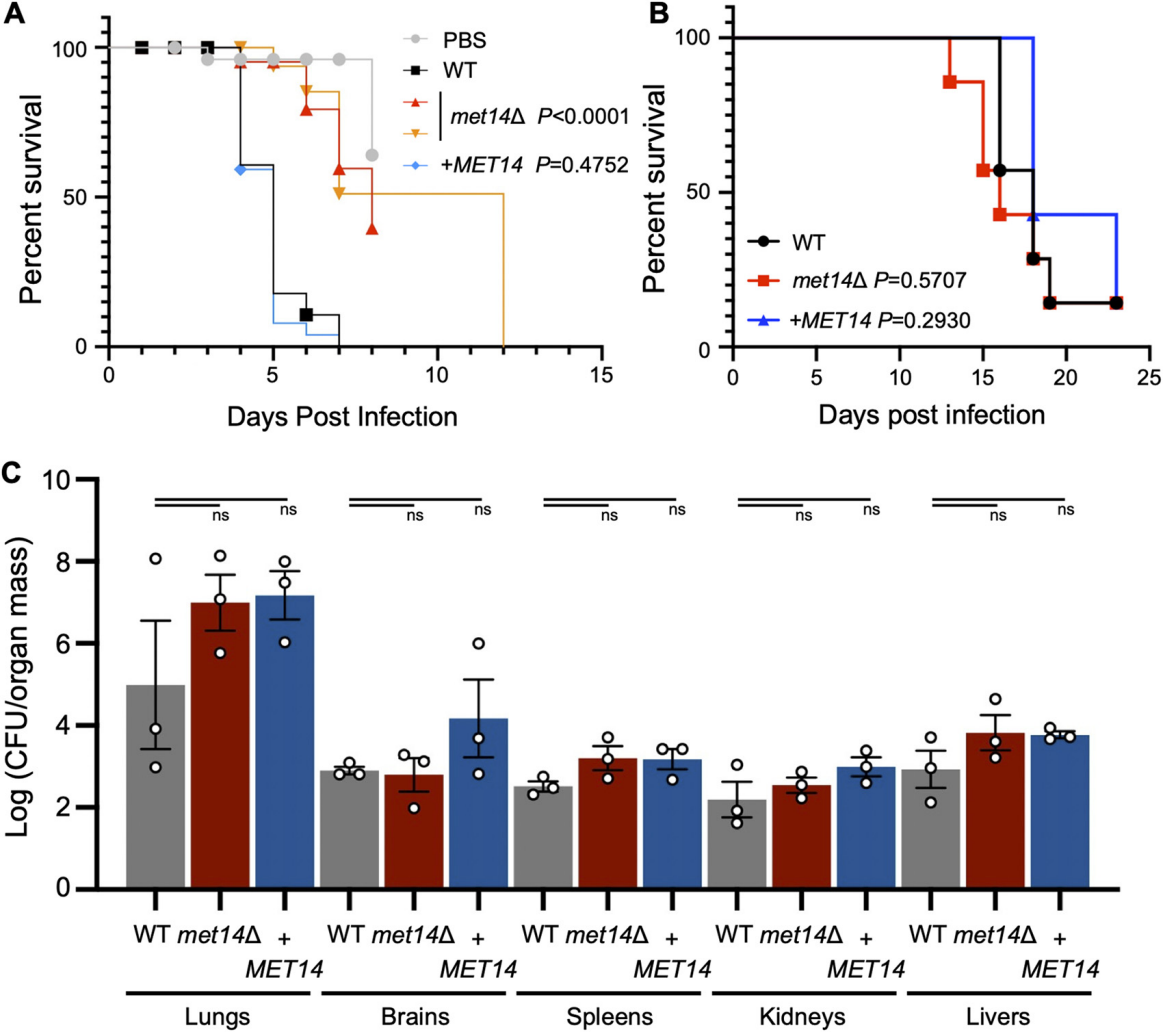
*met14*Δ has attenuated virulence in an insect model, but not in a murine model. (A) Met14 was required for cryptococcal virulence in an insect model. Wild-type, *met14*Δ #1 (YSB6851), *met14*Δ #2 (YSB6852), and complemented strains (YSB10248) were injected into the larvae. Percent survival was monitored for 14 days postinfection. Survival curves were analyzed using the log-rank (Mantel-Cox) test. (B) Met14 was dispensable for cryptococcal virulence in a murine model. Ten BALB/c mice per group were intranasally infected with the wild-type, *met14*Δ (YSB6851), or complemented (YSB10248) strain. The infected mice were monitored daily, and the survival curve was analyzed using the log-rank (Mantel-Cox) test. Sixteen days postinfection, three mice per group were euthanized for the fungal burden assay (C). Fungal burden was calculated by counting the CFU of cryptococcal cells per gram of infected tissue (lungs, brain, spleen, kidneys, and liver). Statistical significance was determined by one-way ANOVA with Bonferroni’s multiple-comparison test using Prism 8.0: *, *P* < 0.1; **, *P* < 0.01; ***, *P* < 0.001; ****, *P* < 0.0001; ns, not significant.

Our findings showed that Met14 is not required for growth of C. neoformans in a nutrient-rich environment, yet it is required for growth and virulence factor production in a nutrient-limited environment and under stress conditions, such as the environment within the host. We thus investigated the pathogenic potential of *met14*Δ in comparison with wild-type and complemented strains by using an insect and a murine model of systemic cryptococcosis. In the insect-killing model using the larvae of Galleria mellonella (27), two independent *met14*Δ mutants showed significantly decreased virulence compared to the wild-type and its complemented strain (Fig. 6A). Hence, Met14 was found to be required for the virulence of C. neoformans, at least in the insect model.

To further assess the pathogenic potential of *met14*Δ in an animal model, we infected mice intranasally with wild-type, *met14*Δ, or a complemented strain and monitored the survival of the infected mice as well as their fungal burden in infected tissues. Surprisingly, in contrast to our findings on the insect model, we found that *met14*Δ was as virulent as the wild-type and complemented strains (Fig. 6B). We also measured the fungal burden in the lungs, brains, spleens, kidneys, and livers recovered from mice at day 16 postinfection. In line with the survival curve, which indicated no change in virulence, the fungal burdens of *met14*Δ also showed no significant differences from those of the wild-type or the complemented strain, indicating that *met14*Δ established systemic cryptococcosis as efficiently as wild-type (Fig. 6C).

### Met3 has Met14-independent pathobiological roles in C. neoformans.

**FIG 7:**
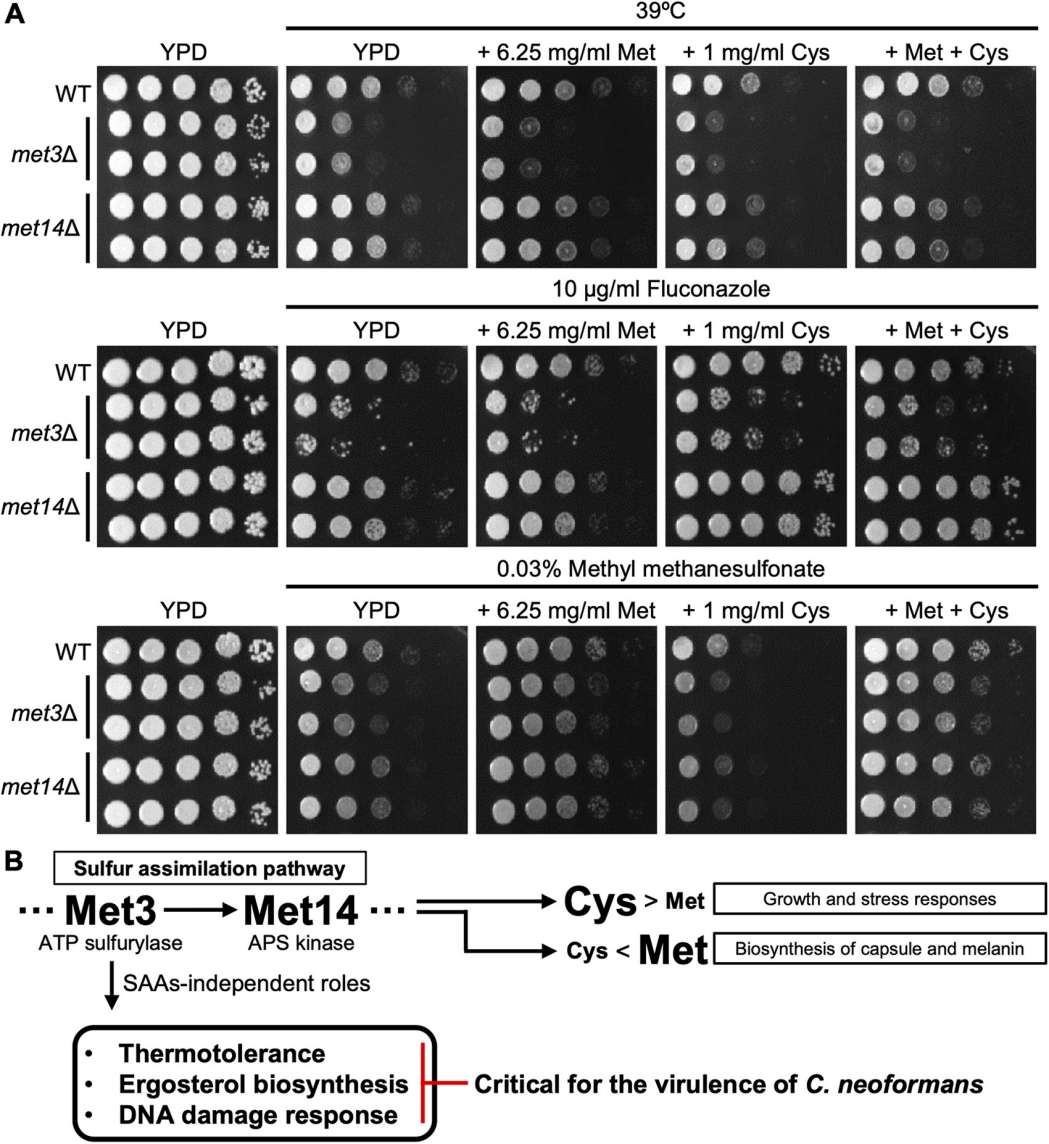
Met3 plays Met14-independent pathobiological roles in C. neoformans. (A) The SAA-independent role of Met3 in thermotolerance, ergosterol biosynthesis, and DNA damage response of C. neoformans. The wild-type (WT; H99), *met3*Δ (YSB3329 and YSB3330), and *met14*Δ (YSB6851 and YSB6852) strains were cultured overnight at 30°C in liquid YPD medium, serially diluted (1 to 10^4^), and spotted onto YPD plates containing 6.25 mg/mL methionine and/or 1 mg/mL cysteine and the indicated amount of stress or antifungal agent. Plates were incubated at 30°C and photographed from 1 to 4 days. (B) The proposed model of Met3- and Met14-dependent pathways in C. neoformans. Met3 and Met14 are required for the synthesis of sulfur amino acids, cysteine and methionine. Cysteine and methionine play major and minor roles, respectively, in the growth and stress responses. In contrast, methionine and cysteine play major and minor roles, respectively, in the production of two major cryptococcal virulence factors, capsule and melanin. Notably, unlike Met14, Met3 plays SAA-independent roles in thermotolerance, ergosterol biosynthesis, and DNA damage response, which are critical for the virulence of C. neoformans.

Our findings indicated that Met14 is dispensable for the virulence of C. neoformans in the murine host; however, this was unexpected, since deletion of Met3, the direct upstream kinase of Met14, was previously found to abolish the virulence of C. neoformans in a murine model of systemic cryptococcosis (15). Therefore, we hypothesized that Met3 may have Met14-independent pathobiological roles in C. neoformans. To test this hypothesis, we compared phenotypic traits of *met14*Δ with those of *met3*Δ with or without SAA supplementation (Fig. 7). All the phenotypic traits of *met14*Δ described above were mostly identical to those of *met3*Δ (Fig. S7). Similar to the case of *met14*Δ, cysteine supplementation was better at restoring the growth and stress and antifungal agent resistance of *met3*Δ than methionine supplementation (Fig. S7A and S7B), whereas methionine supplementation more efficiently restored melanin and capsule production in *met3*Δ (Fig. S7C to S7E). These results suggested that Met3 and Met14 are involved in the SAA biosynthetic pathway.

Despite its redundant roles in the SAA pathway, Met3 also appeared to play Met14-independent roles in C. neoformans. Specifically, we found a higher susceptibility of *met3*Δ to a high temperature level (39°C), the antifungal drug fluconazole, and the DNA-damaging agent methyl methanesulfonate, compared to the wild-type and *met14*Δ strains. These Met14-independent *met3*Δ phenotypes could not be rescued by the addition of SAAs (Fig. 7A). Thermotolerance, ergosterol biosynthesis, and the DNA damage response were previously reported to be important virulence attributes for C. neoformans (28–30), which could explain why Met3, but not Met14, is required for cryptococcal virulence in murine hosts. Collectively, these data indicated that Met3 plays both Met14-dependent and -independent pathobiological roles in C. neoformans.

## DISCUSSION

The sulfate assimilation pathway is found in prokaryotes and certain eukaryotes, such as fungi and photosynthetic plants, and allows the synthesis of sulfur-containing biomolecules, such as sulfur amino acids (e.g., methionine and cysteine) and glutathione from extracellular sulfur sources (31). However, methionine cannot be synthesized *de novo* and therefore must be supplemented into the diet of higher eukaryotes, such as animals and humans (32). Nevertheless, APS kinase, which is the second player in the sulfate assimilation pathway, is present in humans, even though sulfate assimilation does not exist. APS kinase is required for sulfation, which is the reaction usually catalyzed by a sulfotransferase and in which a sulfate group is transferred from PAPS. Sulfation is necessary for various regulatory processes in mammalian cells, such as inactivation or bioactivation of xenobiotics, catecholamines, and hormones, control of macromolecules, and elimination of catabolic end products (33). Humans have two types of APS kinases: PAPS synthetase 1 (PAPSS1) and PAPSS2. PAPSS2 plays significant roles in androgen excess, bone dysplasia, and spondyloepimetaphyseal dysplasia when disrupted (34). PAPSS1 cannot replace PAPSS2 in terms of function, yet only limited information is available in the literature on this kinase type (35).

Sulfur metabolism has been previously reported to regulate virulence of various bacterial and fungal pathogens (36, 37). Therefore, sulfur metabolism has been exploited as a potential target for antimicrobial drugs (38, 39). Although the sulfate assimilation pathway has been extensively studied in many bacterial pathogens (36), only a few studies have been conducted on fungal pathogens. Methionine biosynthesis was reported to be related to the virulence of the most common pathogenic fungi, C. albicans (40). Aspergillus fumigatus methionine biosynthesis has also been highlighted recently as a potential antifungal target (41). In the basidiomycetous pathogenic fungus C. neoformans, methionine and sulfate assimilation pathways were found to be required for the induction of virulence factors and virulence (15, 42, 43). Here, we reason that although amino acid biosynthesis or sulfur metabolism could be exploited as promising antifungal targets, not all components of these pathways are equally relevant for novel antifungal drug discovery.

Here, no growth defects were observed in nutrient-rich YPD medium after deletion of *MET14*, and sulfur-containing compounds in the YPD medium were sufficient to support C. neoformans growth. Cryptococcal *met14*Δ showed methionine and cysteine auxotrophism in minimal media such as YNB. In S. cerevisiae, *met14*Δ is able to grow when only methionine is added to the medium (44). In A. fumigatus, a *metR*Δ strain, which is a mutant of bZIP transcription factor regulating the sulfur assimilation pathway, also shows methionine auxotrophism (13). In the cereal pathogen Fusarium graminearum, Δ*FgMet14* shows methionine and cysteine auxotrophism, and addition of both amino acids rescues the growth defect of this mutant in minimal medium (45). However, we found here that addition of cysteine, but not methionine, could recover the growth of the *met14*Δ mutants of C. neoformans. This finding may be attributed to two reasons. First, unlike S. cerevisiae, A. fumigatus, and F. graminearum, C. neoformans cannot convert methionine to cysteine as efficiently as it converts cysteine to methionine. Second, the uptake of extracellular methionine may be less efficient than that of cysteine. However, the latter possibility is unlikely, since we found that methionine supplementation could restore melanin and capsule production more efficiently than cysteine supplementation. Taken together, these data indicate that cysteine is a better sulfur source than methionine for supporting C. neoformans growth.

Our findings indicated that C. neoformans requires higher levels of intracellular cysteine in nutrient-rich environments under certain stress conditions. The *met14*Δ mutants showed a normal growth profile in nutrient-rich medium (e.g., YPD) yet increased susceptibility to various stress-causing agents. This susceptibility, except that for 5-flucytosine, was fully recovered upon the addition of cysteine, but not methionine. This was further evidenced by recovery of growth of *met14*Δ upon the addition of cysteine in minimal YNB medium supplemented with ammonium sulfate. Hence, extracellular sulfur uptake is crucial for C. neoformans to cope with various environmental stresses. Moreover, the intracellular cysteine concentration is also vital in the response to various stresses. We initially suspected that reduced intracellular glutathione concentrations could contribute to such phenotypes of *met14*Δ. However, the lack of a significant increase in intracellular glutathione concentration upon cysteine addition in both wild-type and *met14*Δ strains suggests that cysteine *per se* is an essential contributing factor. Exogenous methionine, cysteine, and glutathione did not significantly improve the growth of *met14*Δ under the presence of 5-flucytosine, indicating that Met14 is required not only for the synthesis of SAAs but also to prevent DNA and RNA synthesis interference. Even though PAPS is required for sulfation (33, 46), the susceptibility of *met14*Δ toward 5-flucytosine is not likely due to the lack of the intracellular sulfate. Further studies are needed to determine how the APS kinase plays an SAA biosynthesis-independent role in C. neoformans.

Despite the importance of Met14-dependent cysteine synthesis for growth and stress responses, the production of two major cryptococcal virulence factors, the antioxidant melanin pigment and the antiphagocytic polysaccharide capsule, was found to require higher levels of Met14-dependent methionine synthesis than of cysteine synthesis. Melanin and capsule deficiencies observed in *met14*Δ could be more efficiently restored by methionine than by cysteine. Moreover, deletion of Met3, an upstream kinase involved in APS production, was also reported to lead to melanin deficiency in a previous study (15). Here, methionine addition was found to more efficiently rescue this deficiency than cysteine addition. This unexpected finding suggests that SAA uptake is not a major issue in the distinctive contribution of SAAs to the pathobiological functions of C. neoformans. One possible explanation for this is that cysteine-to-methionine conversion might be more efficient than methionine-to-cysteine conversion; however, it is still insufficient to produce sufficient methionine to induce melanin and capsule production. This hypothesis is supported by the recovery of virulence factors at cysteine concentrations that are higher than methionine concentrations. We hypothesize that insufficient intracellular methionine may lead to a lack of *S*-adenosyl-methionine, a versatile methyl donor and modifier of histones, nucleic acids, and phospholipids, owing to an insufficient amount of intracellular methionine and may be the reason behind the specific involvement of methionine in the induction of melanin and capsule production (47, 48). Therefore, methylation-dependent epigenetic regulation is considered critical for melanin production and capsule induction. Further research is needed to fully elucidate this phenomenon.

The most striking and unexpected result of our study was that *met14*Δ exhibited a wild-type pathogenic potential in a murine host model. Previous research suggested that the sulfur metabolic pathway is essential for the virulence of C. neoformans. Our earlier functional survey of C. neoformans kinases indicated that *met3*Δ shows significantly decreased virulence in G. mellonella and has low lung and brain infectivity in a murine infection model (16, 17). Homoserine transacetylase Met2 has also been reported to be involved in methionine biosynthesis in C. neoformans and is required for the virulence (43). The deletion of cryptococcal Met6, which converts homocysteine to methionine, was also found to lead to decreased virulence in mice (42). However, here we demonstrated that although *met14*Δ showed decreased virulence in an insect model, *met14*Δ still exhibited wild-type levels of virulence and fungal burden in the murine model. This may be attributed to the use of a different murine host strain (BALB/c in this study versus A/Jc in other studies). Another plausible explanation is our finding of additional SAA biosynthesis-independent roles of Met3 in thermotolerance, ergosterol biosynthesis, and DNA damage response. All of these Met14-independent Met3 phenotypic traits have been previously reported to be important virulence factors in C. neoformans (28–30). It remains to be elucidated how Met3 is connected to the signaling pathways involved in thermotolerance, ergosterol biosynthesis, and the DNA damage response in C. neoformans.

In conclusion, here we demonstrated that the APS kinase Met14 in the sulfate assimilation pathway is required for SAA biosynthesis and responses against antifungal drugs, ER stress, cell membrane stress, and heavy metals in C. neoformans. Interestingly, the Met14-dependent cysteine was found to play a major role in growth and stress responses of C. neoformans, while the Met14-dependent methionine played a significant role in the production of cryptococcal virulence factors. Interestingly, unlike its upstream kinase Met3, Met14 was not required for the virulence of C. neoformans in a murine infection model. This may be attributed to the more diverse SAA biosynthesis-independent roles of Met3 in thermotolerance, ergosterol biosynthesis, and DNA damage response (Fig. 7B). Thus, targeting the APS kinase is not likely to be an effective antifungal drug development strategy for treating cryptococcosis. Mechanisms of the regulation of fungal pathogen virulence by sulfur metabolism pathways, such as methyl cycle, transsulfuration, OAS pathway, and glutathione biosynthesis pathway, should be addressed in future studies in order to obtain a better understanding in this regard.

## MATERIALS AND METHODS

### Ethics statement.

Animal experiments were performed according to the ethical guidelines of the Institutional Animal Care and Use Committee of Amtix Bio Corp. (ABLAS-003-FO2). The committee approved all the vertebrate studies.

### Strains and media.

C. neoformans strains used in this study are listed in Table S1. Parental and mutant strains were stored as frozen cell stock in 20% glycerol at −80°C. Yeast strains were cultured at 30°C in either a standing incubator (for solid plates) or shaking incubator at 220 rpm (for liquid cultures). The following media were used in this study: yeast extract-peptone-dextrose (YPD) medium (10 g yeast extract, 20 g peptone, and 20 g dextrose per liter) and yeast nitrogen base (YNB) medium without amino acids and ammonium sulfate, which was purchased from Becton, Dickenson and Company. For the melanin production assay, Niger seed medium (35 g Niger seed and 20 g Bacto agar per liter), dopamine medium, and epinephrine medium (1 g l-asparagine, 3 g KH_2_PO_4_, 250 mg MgSO_4_, 1 mg thiamine, 5 μg biotin, and 100 mg l-DOPA or epinephrine hydrochloride per liter) were used. For capsule induction, Littman’s medium was used as previously described (49).

### Construction of the C. neoformans *CTR4* promoter replacement strain, gene deletion mutants, and complemented strains.

In order to replace the native promoter with the copper-regulated *CTR4* promoter, promoter replacement cassettes were constructed as follows: The primer pairs B9342/B9343 and B9344/B9345 were used to amplify the 876-bp 3′ region of the *MET14* promoter and the 765-bp 5′region of the *MET14* exon, respectively. The 2,242-bp *NAT-CTR4* promoter was amplified with the primer pair B354/B355 using pNAT-CTR4 as a template. The *MET14* promoter replacement cassette was generated by double-joint PCR (DJ-PCR) using primer pairs B9342/B1455 and B9344/B1454. The two PCR products were mixed and introduced into C. neoformans wild-type H99 cells via biolistic transformation. Stable transformants were selected on YPD medium containing 100 μg/mL nourseothricin. The transformants were further screened for correct insertion by diagnostic PCR. Finally, Southern blot analysis was performed to validate the correct genotype of the promoter replacement strain. To disrupt *MET14* expression in C. neoformans, primer pairs B2440/B2441 and B2442/B2443 were used to amplify the 5′- and 3′-flanking regions of the *MET14* gene, respectively. The M13Fe and M13Re primers were used to amplify the nourseothricin-resistant selection marker (nourseothricin acetyltransferase [NAT]). Split-gene deletion cassettes were generated by DJ-PCR, as previously described (50), and introduced into C. neoformans H99 via biolistic transformation. Stable transformants were selected on YPD medium containing nourseothricin and screened using diagnostic PCR. Southern blot analysis was performed to confirm the correct genotype of all diagnostic PCR-positive transformants using *MET14*-specific probes. To verify the phenotypic changes of *met14*Δ in C. neoformans H99, complemented strains were constructed as follows. The promoter and open reading frame were PCR amplified with primers B15349 and B15450 and cloned into the pNEO plasmid using a Gibson Assembly master mix (New England Biolabs, Ipswich, MA, USA) to confirm its sequence. The constructed plasmid was linearized with BsmI and then introduced by biolistic transformation into *met14*Δ (YSB6851). Targeted integration of *MET14* was confirmed by diagnostic PCR. The sequences of all primers used are listed in Table S2.

### Growth and chemical susceptibility tests.

C. neoformans cells grown overnight in YPD liquid medium were 10-fold serially diluted (1 to 10^4^) and then spotted on YPD plates, with or without methionine and cysteine, containing chemical agents to induce stress as described previously (51). Type of stresses tested and chemicals used to induce these stress types were as follows: antifungal drug susceptibility (flucytosine and fludioxonil), toxic heavy metals stress (cadmium sulfate [CdSO4]), cell membrane stress (SDS), oxidative stress (hydrogen peroxide, *tert*-butyl hydroperoxide), genotoxic stress (hydroxy urea and methyl methanesulfonate), and ER stress (tunicamycin and dithiothreitol). The spotted plates were incubated at 30°C and photographed from 1 to 5 days after the treatment using a Bio-Rad Gel-doc imaging system.

### Virulence factor induction assays.

Melanin induction assays were performed as previously described (52). Briefly, strains were inoculated into 2 mL of liquid YPD medium and cultured overnight in a shaking incubator at 30°C. Cells were spun down, washed twice with phosphate-buffered saline (PBS), and resuspended in 1 mL PBS. Each strain was spotted (3 μL) on Niger seed agar medium, epinephrine medium, and dopamine medium with a limited glucose concentration (0.1%), to which methionine and cysteine were supplemented, if necessary. The spotted melanin plates were incubated at 37°C and photographed for 1 to 5 days under a microscope (SMZ-168; Motic) at 10× magnification. For the capsule production assay, C. neoformans cells grown overnight in YPD liquid medium were centrifuged and washed twice with PBS. Cell suspensions were spotted (5 μL) on Littman’s medium agar plates and incubated at 37°C for 2 days. After incubation, the capsules were visualized by staining with India ink (Remel Inc., San Diego, CA, USA). Quantitative measurements of capsule thickness were performed by microscopically measuring capsular and cellular diameters using Nikon NIS software, as previously described (53).

### Colorimetric assay for intracellular glutathione quantification.

To measure intracellular glutathione concentrations in cryptococcal cells, a CheKine Micro total glutathione (T-GSH) assay kit (catalog number KTB1670; Abbkine, Wuhan, China) was used. Each tested strain was cultured overnight in 50 mL of liquid YPD medium. Cell cultures were then diluted to an optical density at 600 nm (OD_600_) of 0.2 with fresh liquid YPD medium and further cultured at 30°C in a shaking incubator for 24 h. One milliliter of the cell culture was harvested from each strain, centrifuged, and washed twice with ice-cold PBS. Cells were resuspended in the extraction buffer provided by the manufacturer, homogenized for 30 s using a bead beater (FastPrep-24 5G bead homogenizer, MP Biomedicals), and centrifuged at 8,000 rpm for 10 min. The supernatants were then collected and used for colorimetric quantification of total glutathione following the manufacturer’s protocol. Aliquots of 2 μL of samples were added in a flat-bottom 96-well plate with 18 μL of distilled water. The glutathione standard from the kit was diluted in 10^−1^ extraction buffer and loaded into a 96-well plate at various concentrations. Two microliters of glutathione reductase, 20 μL of glutathione reductase cofactor (the reduced form of nicotinamide adenine dinucleotide phosphate; NADPH) solution, and 140 μL of assay buffer were added to each well. After mixing well, the OD_415_ was measured using a plate reader (iMark microplate reader, Bio-Rad) immediately after adding 20 μL of chromogen, and the plate was further incubated for 30 min at 37°C in dark. After incubation, the OD_415_ was measured again. Thirty milliliters of cultured cells was lyophilized using liquid nitrogen to measure the dry cell weight and normalize the cell concentration.

### Insect-killing virulence assay.

The *in vivo* virulence of wild-type, *met14*Δ, and *met14*Δ::*MET14* complemented strains were examined in an insect model using G. mellonella larvae (ABDragons, Chardon, OH, USA). A cohort of 20 to 30 larvae of the final-instar larvae, with a body weight of 200 to 300 mg, was used per group. Each cryptococcal strain was cultured overnight at 30°C in liquid YPD, harvested, washed three times with PBS, and resuspended in PBS at a concentration of 10^6^ cells/mL. A total of 4 × 10^4^ cells were injected into the second to the last prolegs of each larva using a 100-μL syringe with a 10-μL needle and repeating dispenser (PB600-1, Hamilton Company, Reno, NV, USA). As a negative noninfection control, PBS was injected. Infected larvae were placed in petri dishes and incubated at 37°C in a humidified chamber. Larval survival was monitored daily. The larvae were considered dead when they turned black or showed no movement upon touching. Pupated larvae were censored for statistical analysis, survival curves were drawn using Prism 8.0.1 (GraphPad, San Diego, CA, USA), and log-rank (Mantel-Cox) analysis was performed using the Prism program. We examined two independent *met14*Δ strains and one complemented strain.

### Murine infectivity assay.

The *in vivo* virulence of wild-type, *met14*Δ, and *met14*Δ::*MET14* complemented strains was also assessed in a murine model. Each C. neoformans strain was cultured overnight at 30°C in liquid YPD medium and washed three times with PBS, and its cell concentrations were adjusted to 2.5 × 10^7^ cells/mL in PBS. Seven-week-old BALB/c mice were purchased for this study (JA BIO, Suwon, Republic of Korea). After arrival, the mice were subjected to habituation for a week, and fungal cells were inoculated intranasally; mice were anesthetized with an intraperitoneal injection of Avertin (2,2,2-tribromoethanol), and 5 × 10^5^ cells (20 μL) were injected through intranasal inhalation. Survival was monitored daily, and the mice were euthanized upon reaching humane endpoints. Statistical analysis was performed using log-rank (Mantel-Cox) analysis of the murine survival curve with Prism 8.0.1. Fungal burden analysis of the brain, lungs, spleen, kidneys, and livers was performed on day 16 postinfection. Tissue samples were homogenized with 5 mL of PBS and spread on YPD medium containing 100 μg/mL of chloramphenicol. The plates were incubated at 30°C for 3 days, and the fungal burden (reported in CFU per gram of tissue) was calculated.
