# Supplementary material for: Adenylyl-Sulfate Kinase (Met14)-Dependent Cysteine and Methionine Biosynthesis Pathways Contribute Distinctively to Pathobiological Processes in Cryptococcus neoformans
Source: Microbiol Spectr. 2023 Apr 10;11(3):e00685-23. doi: 10.1128/spectrum.00685-23 (PMC10269642; doi:10.1128/spectrum.00685-23)
Supplement: Supplemental File 1 — Tables S1 and S2 and Fig. S1 to S7. Download spectrum.00685-23-s0001.pdf, PDF file, 2.8 MB [file spectrum.00685-23-s0001.pdf]

SUPPLEMENTAL MATERIAL

Table S1. ***C. neoformans*** **strains used in this study**

Strains	Genotypes	Parents	References
H99	*MAT*α		
YSB3329	*MAT*α *met3*Δ::*NAT-STM*#205	H99	(1)
YSB3330	*MAT*α *met3*Δ::*NAT-STM*#205	H99	(1)
YSB5818	*MAT*α P_{CTR4}:*MET14*::*NAT*	H99	This study
YSB6851	*MAT*α *met14*Δ::*NAT-STM*#122	H99	This study
YSB6852	*MAT*α *met14*Δ::*NAT-STM*#122	H99	This study
YSB10248	*MAT*α *met14*Δ::*MET14*-NEO	YSB6851	This study

References

1. Lee KT, So YS, Yang DH, Jung KW, Choi J, Lee DG, Kwon H, Jang J, Wang LL, Cha S, Meyers GL, Jeong E, Jin JH, Lee Y, Hong J, Bang S, Ji JH, Park G, Byun HJ, Park SW, Park YM, Adedoyin G, Kim T, Averette AF, Choi JS, Heitman J, Cheong E, Lee YH, Bahn YS. 2016. Systematic functional analysis of kinases in the fungal pathogen *Cryptococcus neoformans*. Nat Commun 7:12766.

Table S2. Primers used in this study

Name	Primer description	Sequence (5' to 3')
**B79**	Common diagnostic screening primer	TGTGGATGCTGGCGGAGGATA
**B354**	*NAT-CTR4* primer 1	GCATGCAGGATTCGAGTG
**B355**	*NAT-CTR4* primer 2	GATTGGTGAAGTCGTTGTCG
**B1454**	*NAT* split primer 1	AAGGTGTTCCCCGACGACGAATCG
**B1455**	*NAT* split primer 2	AACTCCGTCGCGAGCCCCATCAAC
**B2440**	*MET14* 5'-flanking region primer 1	AACAACCGAAACCAGCGAC
**B2441**	*MET14* 5'-flanking region primer 2	TCACTGGCCGTCGTTTTACGGAAGGTGATGTTTGTGGC
**B2442**	*MET14* 3'-flanking region primer 1	CATGGTCATAGCTGTTTCCTGCGCCGACAATGGTCTTATC
**B2443**	*MET14* 3'-flanking region primer 2	TCCTGGTCATCGTGCTAACC
**B2444**	Screening primer for P_{CTR4}:*MET14* and *met14*Δ, pairing with B79	CTTATGCCACTCCTAACCG
**B2445**	*MET14* Southern blot probe primer, paring with B2440	GCCGAGATACCTGTAAAGTCC
**B9342**	*MET14* promoter region primer 1 / Sequencing primer for *MET14* complementation	TAACAACCGAAACCAGCG
**B9343**	*MET14* promoter region primer 2 / Sequencing primer for *MET14* complementation	CACTCGAATCCTGCATGCTTTTTTTTATTTATGGGT
**B9344**	*MET14* exon region primer 1	CGACAACGACTTCACCAATCATGGCCACAAACATCACC
**B9345**	*MET14* exon region primer 2	ATTCGCAGGAGCCTCATAC
**B15349**	LP for *MET14* complementation	GAAGGGCGAATTCTGCAGATATCAATACAGCCCGAACCAGAG
**B15350**	RP for *MET14* complementation	CGGCCGCCAGTGTGATGGATATCTCATTTCTGCCCCATTCC
**B15462**	Screening primer for *MET14* complementation	GCTCTTATGCCACTCCTAAC
**B4272**	Sequencing primer for *MET14* complementation	CCTCCGACAACCATACACTC
**B17753**	*MET14* qRT-PCR primer 1	TCCCGAGTGGAAAACATTC
**B17754**	*MET14* qRT-PCR primer 2	GAGTTGTCGGTCAGAGATG
**B679**	*ACT1* qRT-PCR primer 1	CGCCCTTGCTCCTTCTTCTATG
**B680**	*ACT1* qRT-PCR primer 2	GACTCGTCGTATTCGCTCTTCG
**M13Fe**	M13 forward-extended primer / Sequencing primer for *MET14* complementation	GTAAAACGACGGCCAGTGAGC
**M13Re**	M13 reverse-extended primer	CAGGAAACAGCTATGACCATG

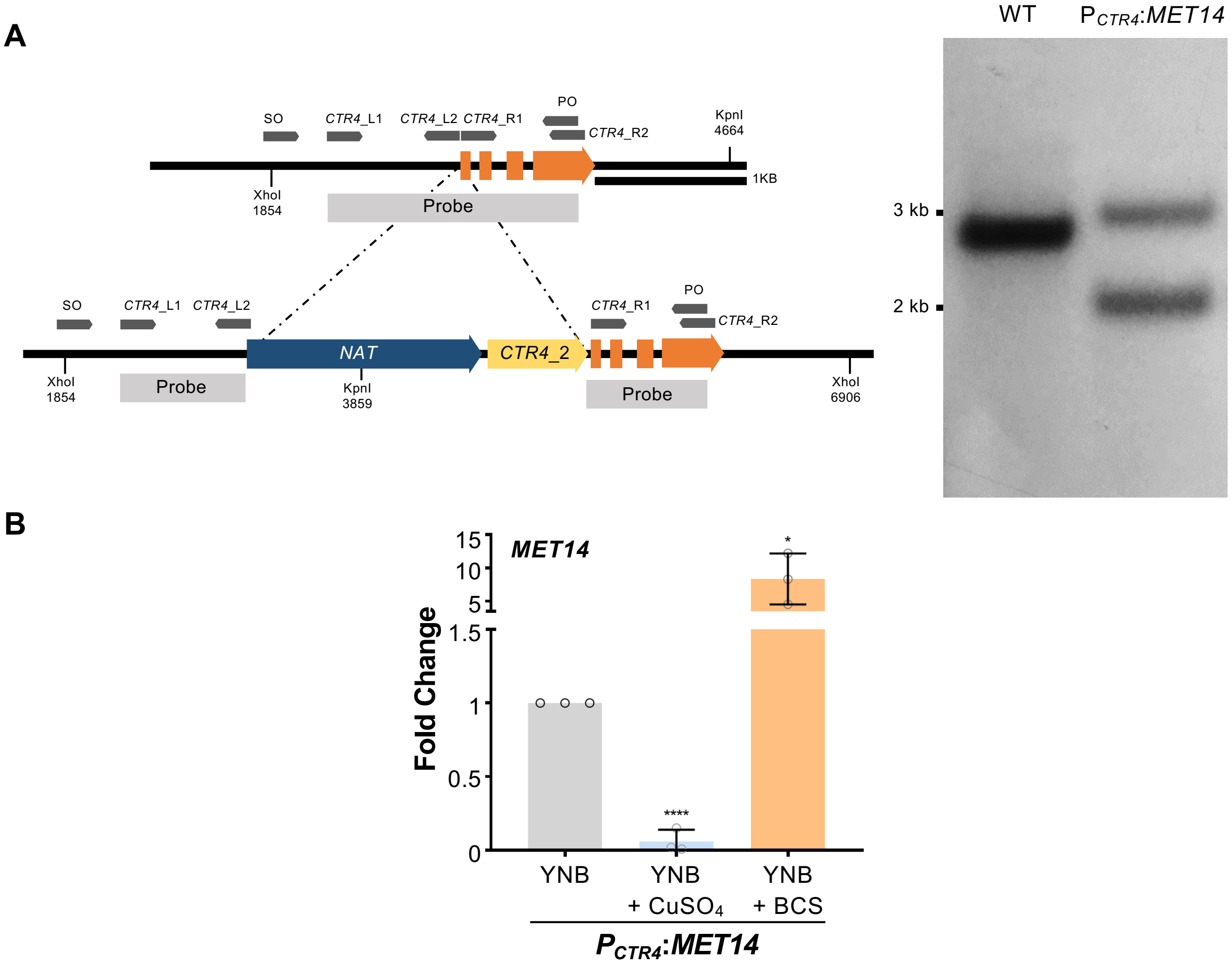

Figure S1. Construction of the P$_{CTR4}$:*MET14* promoter replacement strain. (A) Schematic strategy for the construction of *CTR4* promoter replacement and the result of Southern blot analysis. Correct genotype of P$_{CTR4}$:*MET14* was confirmed through Southern blot analysis with KpnI/XhoI-digested genomic DNA and the *MET14*-specific probe that was PCR amplified with primers B2440 and B2444. (B) Quantitative reverse transcription-PCR (qRT-PCR) of the controlled *MET14* expression by the *CTR4* promoter. P$_{CTR4}$:*MET14* (YSB5818) was grown overnight at 30℃ in 50 ml of liquid YPD medium, subcultured into fresh YPD medium, and further incubated at 30℃ for ~4 h until the culture reached OD$_{600nm}$ of 0.6. Cell culture was separated into three equal volumes, spun down, washed twice with PBS, and resuspended into 50 ml of fresh YNB medium, YNB medium containing 25 μM of $CuSO_4$, or YNB medium containing 200 μM of BCS, and further incubated at 30℃ in a shaking incubator for 24 h. Cell cultures were frozen in liquid nitrogen and lyophilized. Total RNAs were isolated by easy-BLUE™ total RNA isolation extraction kit (iNtRON Biotechnology, Seongnam-si, Republic of Korea). The cDNA was synthesized using Maxima H minus reverse transcriptase (Thermo Fisher Scientific, MA, USA). qRT-PCR was performed using *MET14*-specific primers B17753 and B17754. Gene expression levels of *MET14* were normalized with those of *ACT1*. Statistical significance of difference was determined by one-way ANOVA with Bonferroni's multiple-comparison test using Prism 8.0; * $P < 0.1$; ** $P < 0.1$; *** $P < 0.001$; **** $P < 0.0001$; ns: not significant.

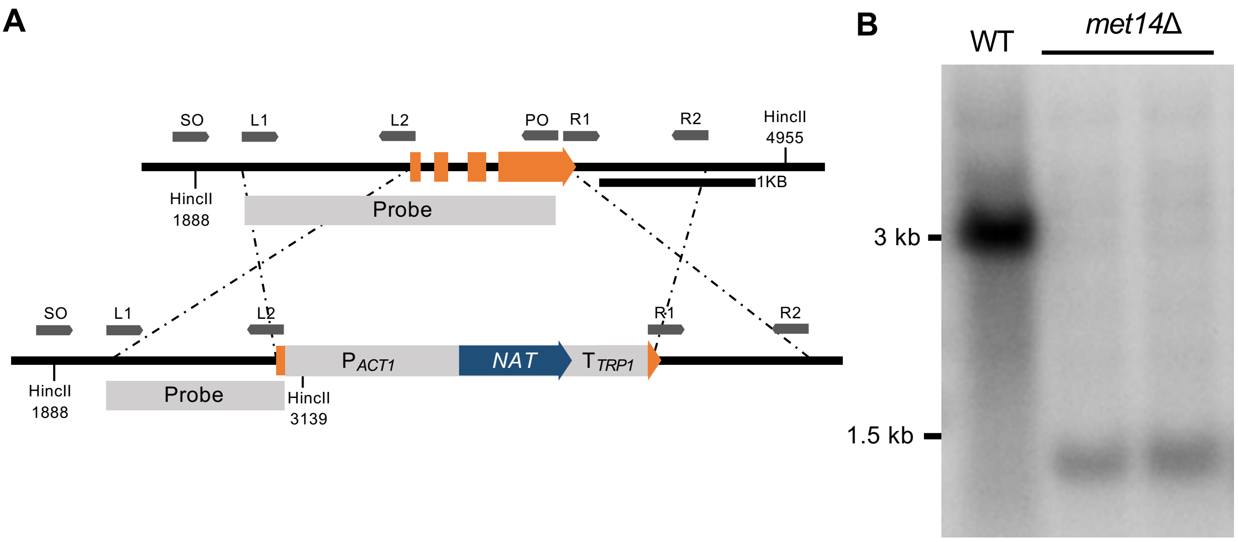

Figure S2. Construction of *met14*Δ mutants. (A) The scheme for the construction of *met14*Δ mutant. (B) The correct genotypes of *met14*Δ mutants (YSB6851 and YBS6852) were confirmed by Southern blot analysis with HincII digested genomic DNAs and the *MET14*-specific probe that was PCR-amplified with primers B2440 and B2444.

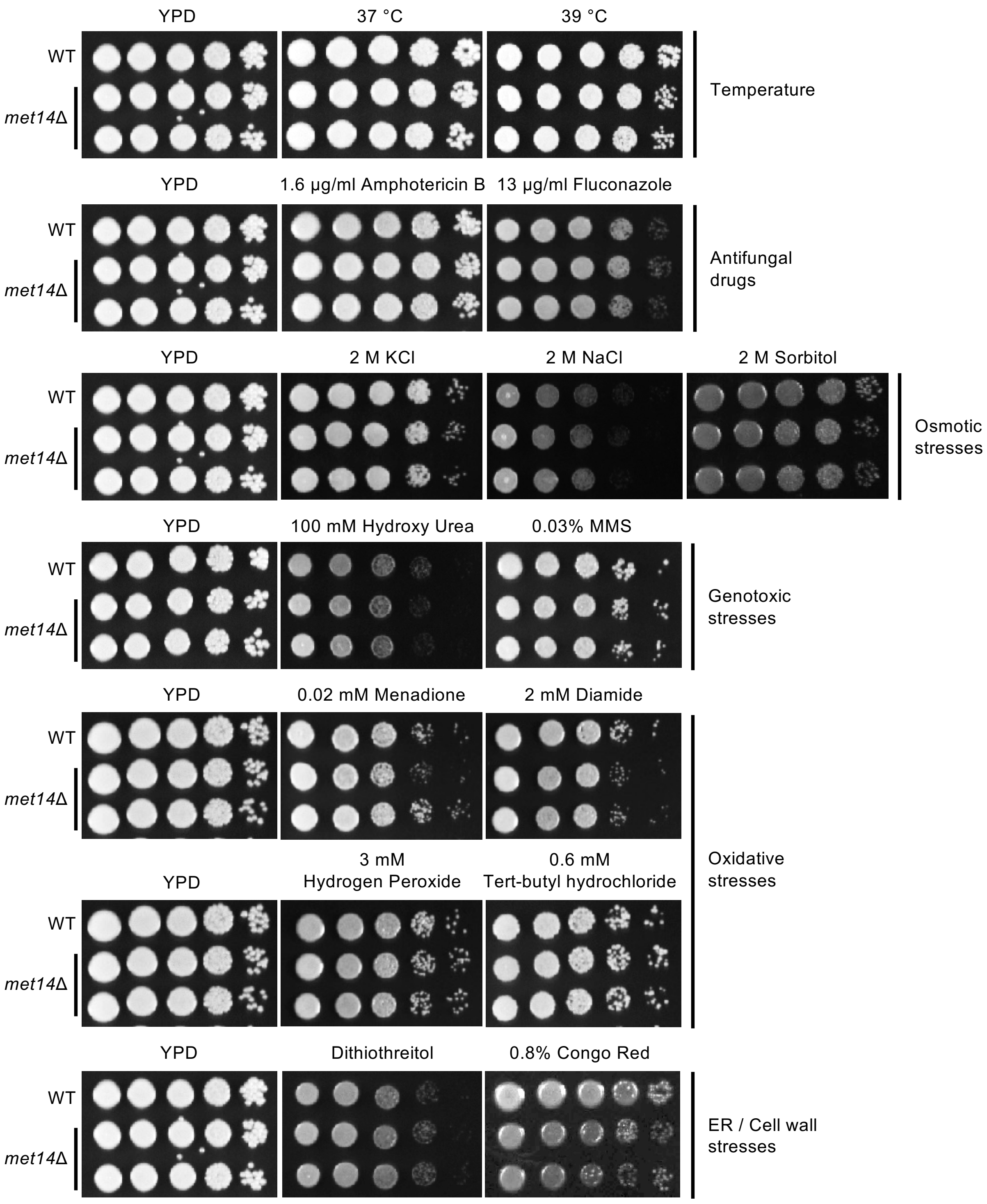

Figure S3. The *met14*Δ mutant grew similar to WT at high temperatures (37℃ and 39℃), and in the presence of antifungal drugs, osmotic stresses, genotoxic stresses, oxidative stresses and ER/Cell wall stresses. The wild-type (WT; H99) and *met14*Δ (YSB6851 and YSB6852) were cultured overnight at 30℃ in liquid YPD medium, serially diluted (1 to 10^4), and spotted onto the YPD plates containing 6.25 mg/ml methionine and/or 1 mg/ml cysteine with the indicated amount of stress or antifungal agents. Plates were incubated at 30℃, and photographed from day 1 to 4.

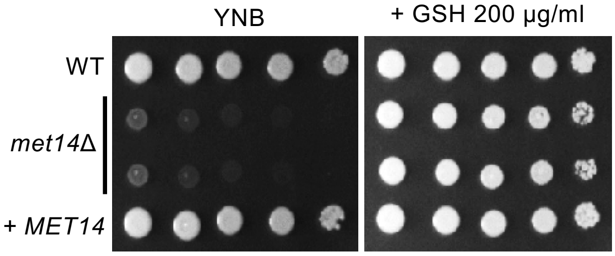

Figure S4. Glutathione (GSH) rescued the growth of *met14*Δ. The wild-type (WT; H99), *met14*Δ (YSB6851 and YSB6852) and *met14*Δ::*MET14* (YSB10248) complemented strain were cultured overnight at 30℃ in liquid YPD medium, serially diluted (1 to 10^4), and spotted onto the YNB plates containing 200 μg/ml of GSH. Plates were wrapped in aluminum foil, incubated at 30℃, and photographed on day 2.

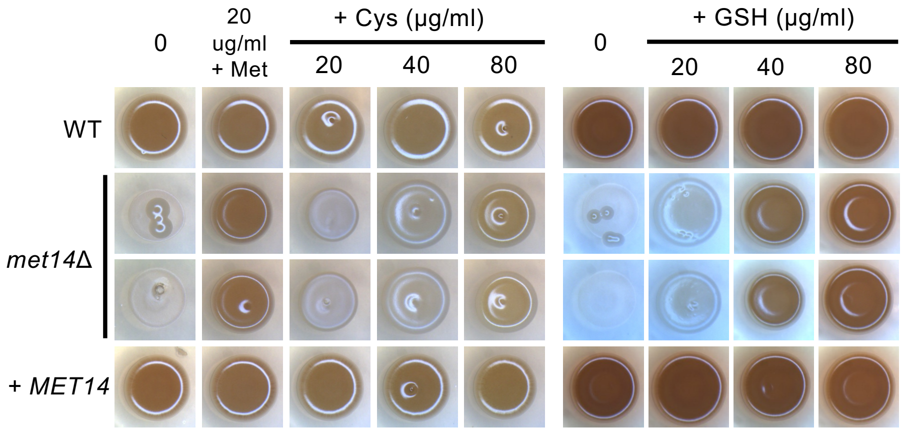

Figure S5. Met14-dependent methionine synthesis is more important for the production of melanin pigment in epinephrine medium than cysteine synthesis. Wild-type (WT; H99), *met14*Δ (YSB6851 and YSB6852) and *met14*Δ::*MET14* (YSB10248) complemented strains were cultured overnight in 30℃ in liquid YPD, washed twice with PBS and spotted (3 µl) onto the plates containing indicated amount of methionine, cysteine, or glutathione. Plates were incubated at 37℃, and spots were photographed on day 3.

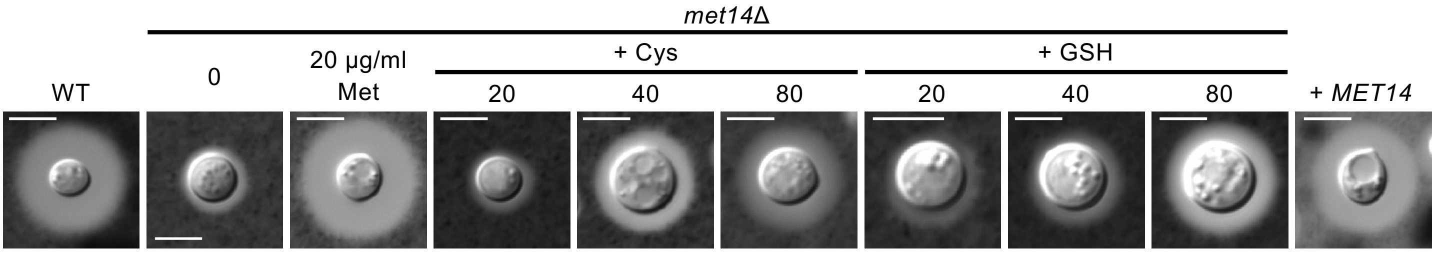

Figure S6. Representative microscopic images of *met14*Δ after capsule induction on Littman's medium (Scale bar = 5 µm). Indicated amounts of methionine, cysteine or glutathione were added to the medium.

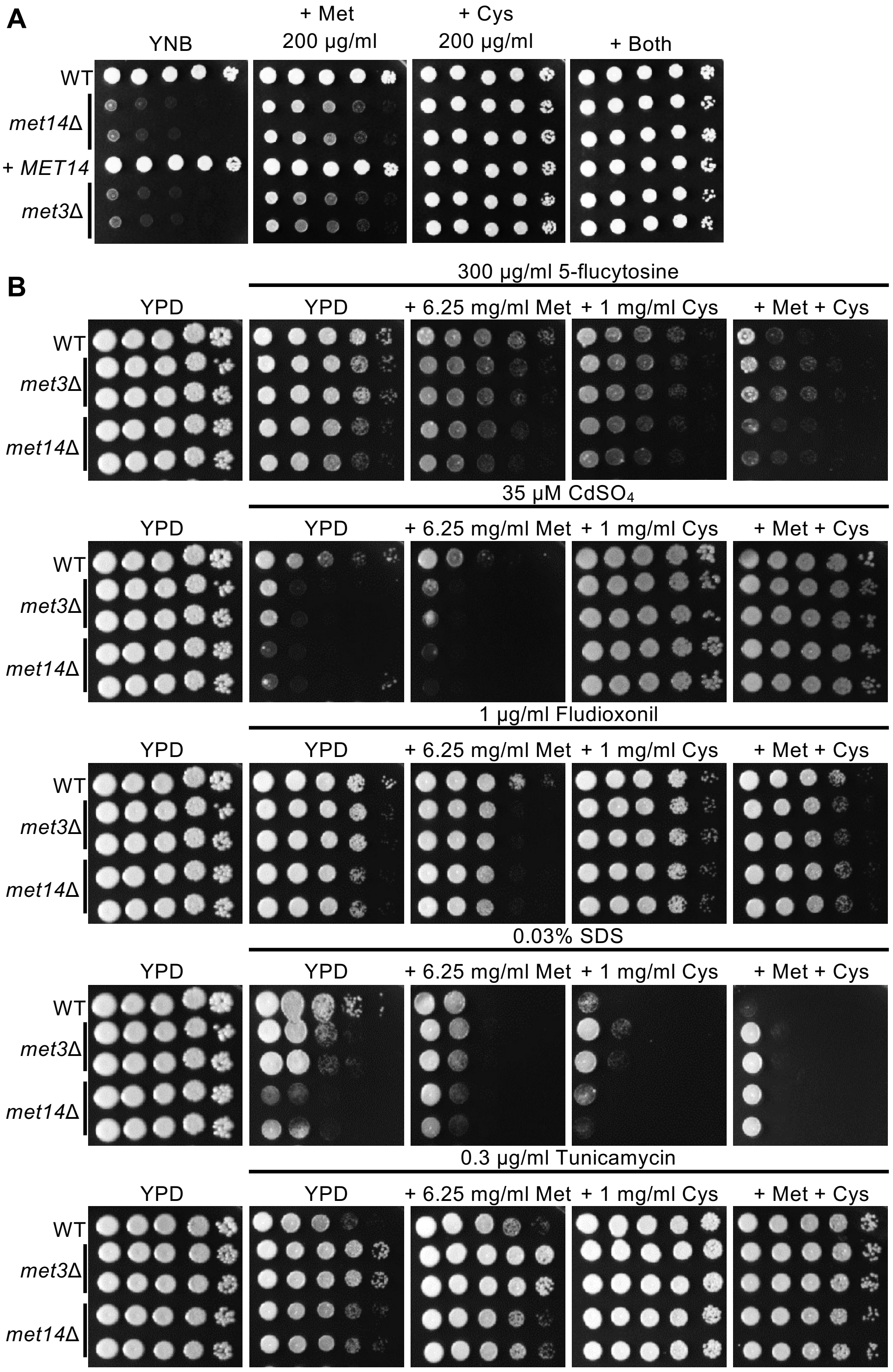
A
YNB
+ Met 200 μg/ml
+ Cys 200 μg/ml
+ Both
WT
met14Δ
+ MET14
met3Δ
B
300 μg/ml 5-flucytosine
35 μM CdSO4
1 μg/ml Fludioxonil
0.03% SDS
0.3 μg/ml Tunicamycin
YPD
+ 6.25 mg/ml Met
+ 1 mg/ml Cys
+ Met + Cys
WT
met3Δ
met14Δ

Continued

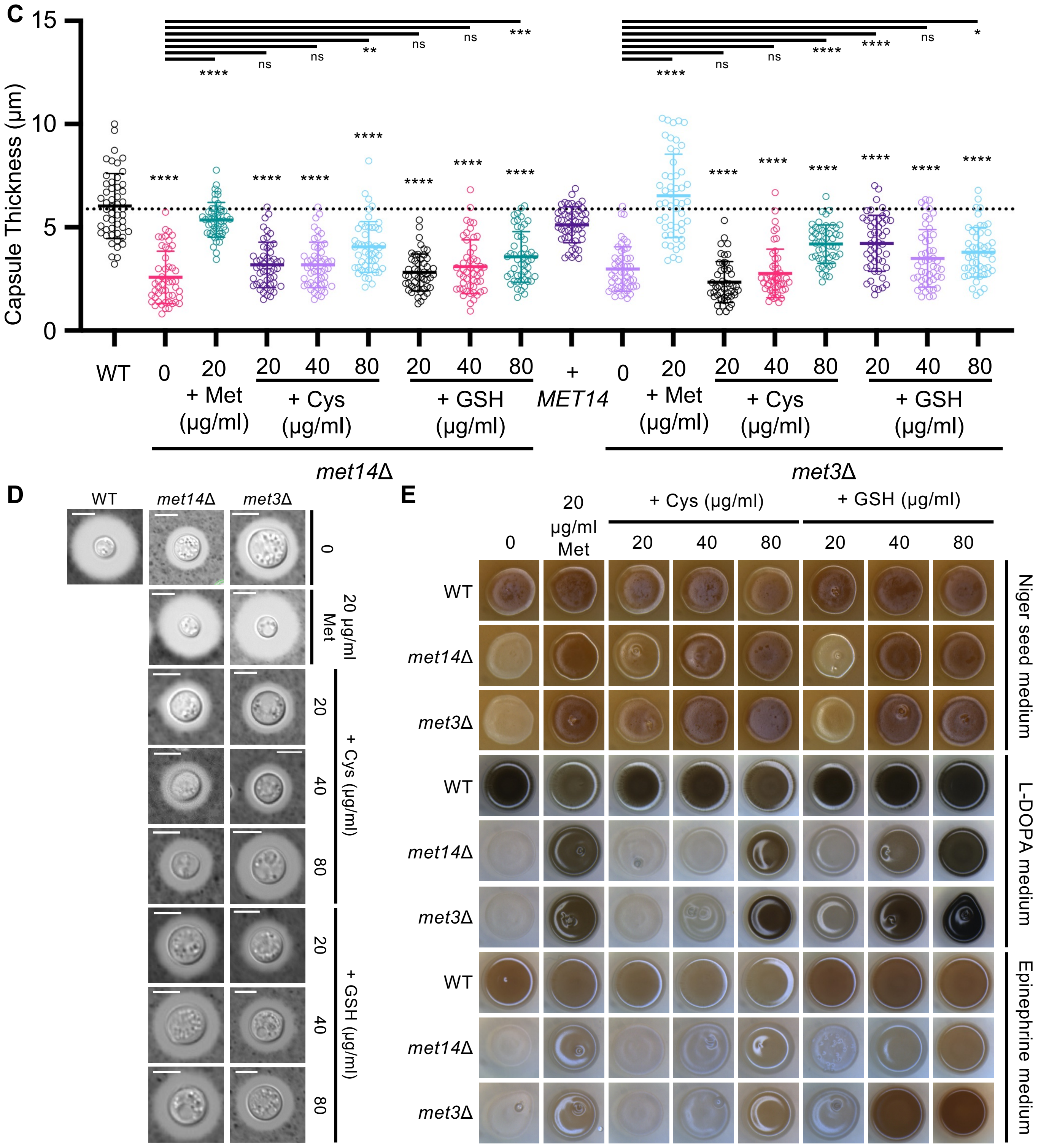

Figure S7. Met3 and Met14 in the SAA biosynthetic pathway play redundant roles in the growth, stress response, and virulence factor production of *C. neoformans.* (A) Methionine- and cysteine-auxotrophic growth of *met3*Δ. The wild-type (WT; H99), *met3*Δ (YSB3329 and YSB3330) and *met14*Δ (YSB6851 and YSB6852) were cultured overnight at 30℃ in liquid YPD medium, serially diluted (1 to 10^4), and spotted onto the YNB plates containing indicated concentration of SAAs. Plates were incubated at 30℃ and photographed on day 2. (B) Met3-dependent stress phenotypes in *C. neoformans*. The wild-type (WT; H99), *met3*Δ (YSB3329 and YSB3330) and *met14*Δ (YSB6851 and YSB6852) were cultured overnight at 30℃ in liquid YPD medium, serially diluted (1 to 10^4), and spotted onto the YPD plates containing 6.25 mg/ml methionine and/or 1 mg/ml cysteine with the indicated amount of stress or antifungal agents. Plates were incubated at 30℃ and photographed from 1 to 4 days. (C) Capsule induction assay in Littman's medium supplemented with the indicated amount of methionine, cysteine and glutathione. Each measurement was repeated for 50 cells in each condition as indicated. Error bars indicate standard deviation, and statistical significance of difference was determined by one-way ANOVA with Bonferroni's multiple-comparison test using Prism 8.0; * $P < 0.1$; ** $P < 0.1$; *** $P < 0.001$; **** $P < 0.0001$; ns: not significant. (D) Representative microscopic images of *met3*Δ compared to *met14*Δ after capsule induction on Littman's medium (Scale bar = 5 μm). Indicated amounts of methionine, cysteine or glutathione were added to the medium. (E) Melanin induction assay of *met3*Δ compared to *met14*Δ on niger seed, L-DOPA and epinephrine medium. Indicated amounts of methionine, cysteine or glutathione were added to the medium.
